# ﻿*Lysimachiadanxiashanensis*, a new species of Primulaceae from Guangdong, China

**DOI:** 10.3897/phytokeys.237.114484

**Published:** 2024-01-31

**Authors:** Xing-Yue Zhang, Jing-Min Dai, Qiang Fan, Zai-Xiong Chen, Guang-Da Tang, Wen-Bo Liao

**Affiliations:** 1 State Key Laboratory of Biocontrol and Guangdong Provincial Key Laboratory of Plant Resources, School of Life Sciences, Sun Yat-sen University, Guangzhou 510275, China; 2 National Park and Nature Education Research Institute, Sun Yat-sen University, Guangzhou 510275, China; 3 Administrative Commission of Danxiashan National Park, Shaoguan 512300, China; 4 College of Forestry and Landscape Architecture, South China Limestone Plants Research Center, South China Agricultural University, Guangzhou 510642, China

**Keywords:** Danxia landscape, IUCN Red List, *Lysimachia*, taxonomy

## Abstract

*Lysimachiadanxiashanensis*, a new Primulaceae species, endemic to the Danxia landscape in Guangdong Province, China, is described and illustrated. This new species is morphologically similar to *L.pseudohenryi*, *L.phyllocephala*, *L.congestiflora* and *L.kwangtungensis*, but it differs from the similar species by its purplish-red plants, petiole without wings, calyx with orange glandular and the corolla margin serrated on upper half with orange-red glandular punctates. This new species belongs to Lysimachiasubgen.Lysimachiasect.Nummularia. Phylogenetic analysis confirmed that *L.danxiashanensis* is a distinct clade, based on the combined data of ITS and *rbc*L sequences. The conservation status of the new species was evaluated as Endangered (EN) according to IUCN Red List Categories and Criteria.

## ﻿Introduction

*Lysimachia* L. is a member of the tribe Lysimachieae (Primulaceae) and is composed of over 200 species (Chen and Hu 1979; Hu and Kelso 1996; Wang et al 2018). This genus is the most widely distributed genera of Primulaceae and is mainly distributed in the temperate and subtropical regions of the Northern Hemisphere, but there are also a few species in Africa, Australia and South America (Hu 1994; Hao and Hu 2001; Hao et al. 2004; Kodela 2006). China is considered to be the origin and diversity centre of *Lysimachia*, with 138 native species having been recorded in Flora of China and nearly 80% of them are endemic species (Chen and Hu 1979; Hu and Kelso 1996; Hao and Hu 2001). In recent years, many new species of this genus have been described and this highlights more opportunities for discoveries in China (Wang et al 2018; Huang et al 2020; Yan et al 2022).

An unknown species of *Lysimachia* was discovered during a field floristic investigation from May 2022 to August 2023 in Danxiashan National Park, Renhua County, Guangdong Province. It is most similar to *L.congestiflora* Hemsl., but its purplish-red plants, petiole without wings, corolla lobes serrated on upper half and calyx with orange glandular punctates clearly distinguish from the latter. After careful morphological comparison by specimens and consultation with relevant literature and molecular phylogenetic analysis, we confirmed that it represented a new species, described and illustrated here. The threat status of the new species is assessed according to the IUCN Red List Categories and Criteria (IUCN Standards and Petitions Committee 2022).

## ﻿Materials and methods

### ﻿Morphological study

The morphological characters of the new species were observed and measured, based on fresh and dry specimens using a micrometer and a stereomicroscope and were compared with its related species, based on herbarium specimens deposited at the Herbarium of SYS and IBSC (the herbarium acronyms follow Thiers (2023)), as well as the digital images on the Chinese Virtual Herbarium (https://www.cvh.ac.cn/) and the China Field Herbarium (https://www.cfh.ac.cn/). Morphological observation and examination were conducted in the SYS.

### ﻿Taxon sampling and molecular analysis

Leaf tissue of the putative new species and related species was collected from one population and silica dried in zip-lock plastic bags until use for comparisons and taxonomic treatment. Total DNA was extracted with a modified CTAB method (Doyle and Doyle 1987). Regions of the partial internal transcribed spacer 1, the 5.8S ribosomal RNA gene and partial internal transcribed spacer 2 were amplified using the previously-reported primers ITS1 and ITS4 (White et al. 1990) and the ribulose-1,5-bisphosphate carboxylase/oxygenase large subunit (*rbc*L) gene was amplified using the primers *rbc*La-f (Kress and Erickson 2007) and 724R (Fay et al. 1997). PCR amplifications were performed following Fan et al. (2015). Following the studies of *Lysimachia* (Zhang et al. 2011; Yan et al. 2018), we retrieved 67 ITS and *rbc*L accession of 30 species from GenBank, which belong to subgenus LysimachiaL.,subgenusPalladia (Moench) Hand.-Mazz., subgenus Heterostylandra (Hand. -Mazz.) Chen et C.M.Hu. and subgenus Idiophyton Hand.-Mazz. Two accessions of the putative new species (GenBank Acc. ITS No.: OR665389, OR665390; *rbc*L No: PP025352, PP035354) and one accession of *Lysimachiakwangtungensis* (GenBank Acc. ITS No.: OR941025; *rbc*L No: PP025355) were sequenced for this study. *Ardisiaverbascifolia* was selected as outgroup. The sequences were aligned using MAFFT v.7 (Katoh and Standley 2013) and subsequently manually adjusted. Phylogenetic constructions were based on Maximum Likelihood (ML) and Bayesian Inference (BI) and were respectively run by IQ-TREE v. 2.0.3 (Minh et al. 2020) and MrBayes version 3.1.2 (Huelsenbeck and Ronquist 2001), selecting best-fit model as SYM+I+G4 with 2000 bootstraps (BS) for ML analysis. ModelFinder v.2.2.0 (Kalyaanamoorthy et al. 2017) was used to select the best-fit partition model (Edge-linked) using the BIC criterion. The best-fit models according to BIC were SYM+G4 for ITS and K2P+I+G4 for *rbc*L. BI analysis employed random starting trees and four Markov Chain Monte Carlo (MCMC) simulations were run simultaneously and sampled every 1000 generations for 10 million generations. The average standard deviation of split frequencies (< 0.01) was used to assess the convergence of the two runs. Bayesian posterior probabilities (PP) were calculated as the majority consensus of all sampled trees with the first 25% discarded as burn-in.

## ﻿Results and discussion

### ﻿Morphological comparison

According to the classification of Chen and Hu (1979), *L.danxiashanensis* is a member of subgenus Lysimachiasect.Nummularia, which is characterised by stems prostrate to erect on the upper part, leaves opposite, racemes shortened to subcapitate, filaments longer than anthers, lower part connate into a tube, corolla and calyx with coloured glandular punctates (Fig. 2). In China, there are over 50 species of sect. Nummularia and it widely distributed from southwest to the east and south China. Morphologically, *Lysimachiadanxiashanensis* is similar to *L.phyllocephala* Hand.-Mazz., *L.pseudohenryi* Pamp., *L.congestiflora* Hemsl. and *L.kwangtungensis* (Hand.-Mazz.) C.M.Hu by sharing the following morphological features: stems with multicellular hairs, leaves opposite and racemes terminal. However, the new species can be easily distinguished from similar species by combination characters including its purplish-red plants (vs. green), petiole without wings (vs. narrowly winged in *L.pseudohenryi* and *L.congestiflora*, narrowly margined and auriculate at base in *L.kwangtungensis* and absent in *L.phyllocephala*) and the corolla lobes serrated on upper half (vs. entire margin in all four species). A more detailed morphological comparison of these species is summarised in Table 1.

**Table 1.** Morphological comparison of *Lysimachiadanxiashanensis* with its four closely-related species.

Characters	*L.danxiashanensis*	*L.phyllocephala*	*L.pseudohenryi*	*L.congestiflora*	*L.kwangtungensis*
Stems	upper erect, creeping at base	erect to ascending-erect, prostrate at base	erect or arcuate at base	prostrate and branches ascending	erect
Colour of plants	purplish-red	green	green	green	green
Leaf shape	ovate to broadly ovate or oval	ovate to ovate-lanceolate	rhomboid-ovate to ovate, rarely ovate-lanceolate	ovate to broadly ovate or suborbicular	ovate-lanceolate to lanceolate
Petiole wings	absent	absent	narrowly winged	narrowly winged	narrowly margined and auriculate at base
Corolla lobes	margin serrated on upper half with orange-red glandular punctates	margin entire, with sparsely transparent glandular punctates	margin entire, with transparent glandular punctates	margin entire, with dull red or black glandular punctates	margin entire, with red to dark purple glandular punctates
Style	6–8 mm; glabrous	*c.* 8 mm; puberulous	5–6 mm; lower part with pubescent	5–7 mm; glabrous	5–6 mm; glabrous
Glandular dots on Calyx	orange, dense	absent	absent	absent	orange, sparse

### ﻿Molecular analysis

**Figure 1.**
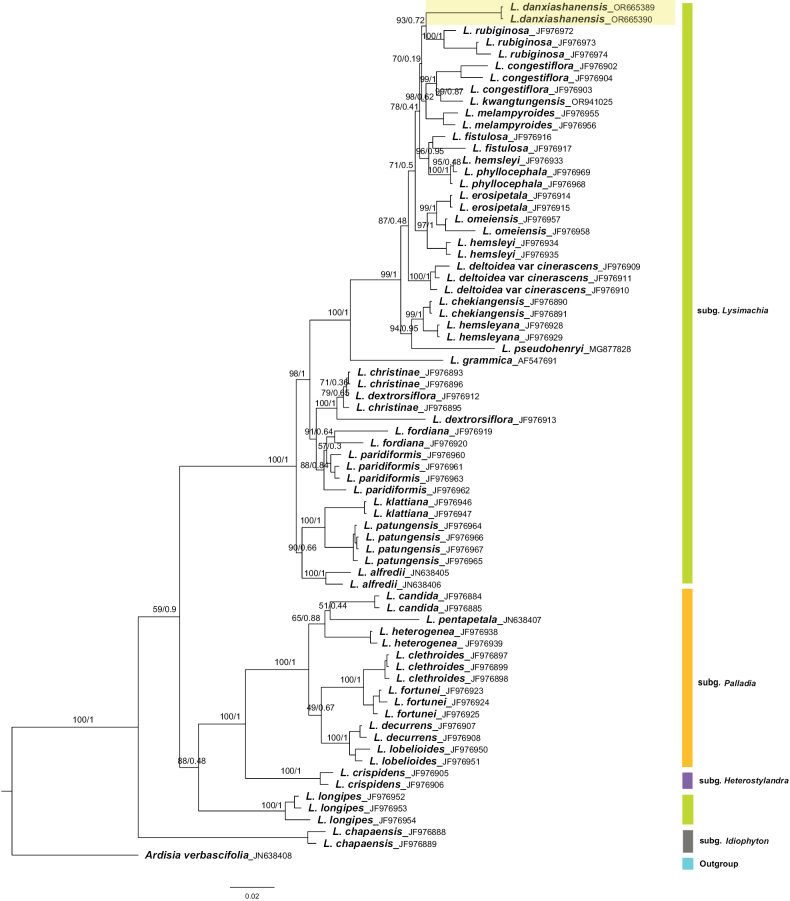
Phylogenetic tree of *Lysimachiadanxiashanensis* and related species generated by Bayesian Inference (BI) of the combined dataset (ITS, *rbc*L). Bootstrap values of the ML and BI posterior probabilities are shown along the branches. The new species in yellow shaded area, green indicates subgen. Lysimachia, orange is subgen. Palladia, purple is subgen. Heterostylandra and grey is subgen. Idiophyton. Blue indicates outgroup, *Ardisiaverbascifolia*.

The combined aligned matrix had a length of 1268 bp (ITS: 650, *rbc*L: 615), including 373 variable sites, of which 291 were parsimony-informative. The two accessions of the new species were from the same population and formed a separate monophyletic lineage (Fig. 1: BS = 93%, PP = 0.72), the sister group of *L.rubiginosa*. Although *L.danxiashanensis* and *L.rubiginosa* both belong to subgen. Lysimachiasect.Nummularia, the new species can be easily distinguished from the latter by its shorter plants (10–28 cm vs. 30–60 (100) cm), orange glandular punctates on corolla lobes and calyx (vs. black or brown glandular striate on leaves, corolla lobes and calyx), 5–9 flowered on branches and stems axis (vs. 3–5 flowered on branches, seldom on main axis).

Geographically, *L.rubiginosa* is distributed in Guangxi, Guizhou, Hubei, Hunan, Sichuan, Yunnan and Zhejiang and it mainly grows in limestone. In contrast, the new species is distributed in Danxia landscape, Guangdong. The geographical distribution of these two taxa does not overlap.

Although the infrageneric phylogenetic relationships within Chinese *Lysimachia* remain controversial (Zhen and Chen 2012; Liu et al. 2023), the phylogenetic tree placed *L.danxiashanensis* distant from other species in this genus (Fig. 1). Based on the morphological and molecular evidence, we confirmed that *L.danxiashanensis* is a distinct species. Therefore, we describe and provide illustrations for the new species below.

## ﻿Taxonomic treatment

### ﻿ Lysimachia danxiashanensis

Taxon classificationPlantaeEricalesPrimulaceae

W.B.Liao, Q.Fan & G.D.Tang sp. nov.

07E770A5-FE9F-5DF7-82E0-E4297301B023

urn:lsid:ipni.org:names:77335469-1

Figs 2 , 3

#### Diagnosis.

*Lysimachiadanxiashanensis* can be distinguished from *L.congestiflora* by its purple-red plants (vs. green), petiole without wings (vs. narrowly winged), corolla lobes yellow with serrations on upper half (vs. dull red at base with entire margin) and calyx with orange glandular (vs. without glandular) (Fig. 4).

**Figure 2.**
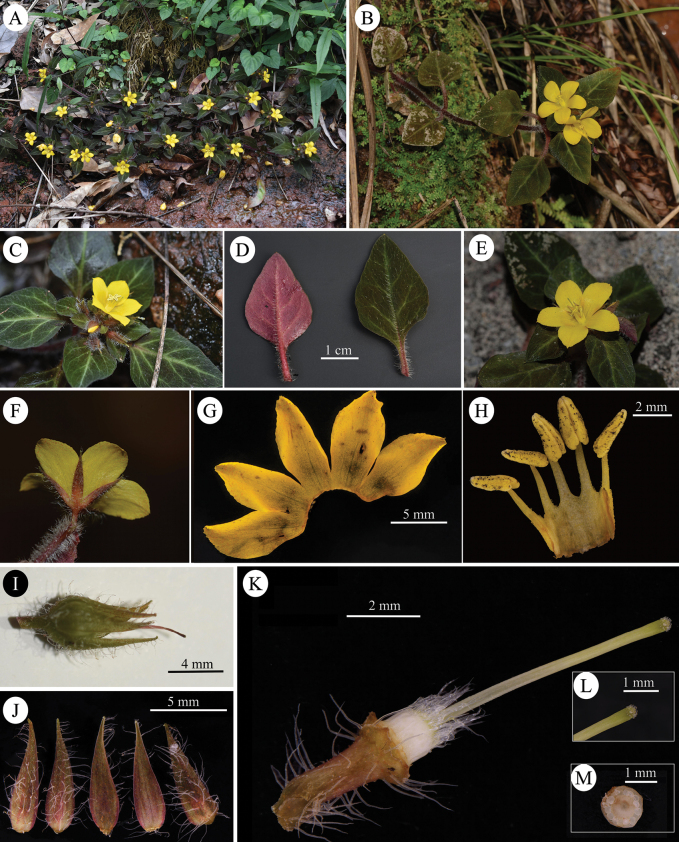
*Lysimachiadanxiashanensis* W.B.Liao, Q.Fan & G.D.Tang, sp. nov. **A** habit **B** flowering branch **C** inflorescence **D** abaxial and adaxial views of leave **E** lateral view of flower **F** dorsal view of flower **G** adaxial side of corolla lobes **H** stamens **I** immature capsule **J** abaxial (1^st^, 2^nd^, 5^th^) and adaxial (3^rd^, 4^th^) views of calyx lobes **K** pistil and densely pilose pedicel **L** stigma **M** cross-section of ovary (Photographers: **A, C, D, I** by Xing-Yue Zhang; **B, E, F** by Qiang Fan; **G, H, J–M** by Jing-Min Dai).

**Figure 3.**
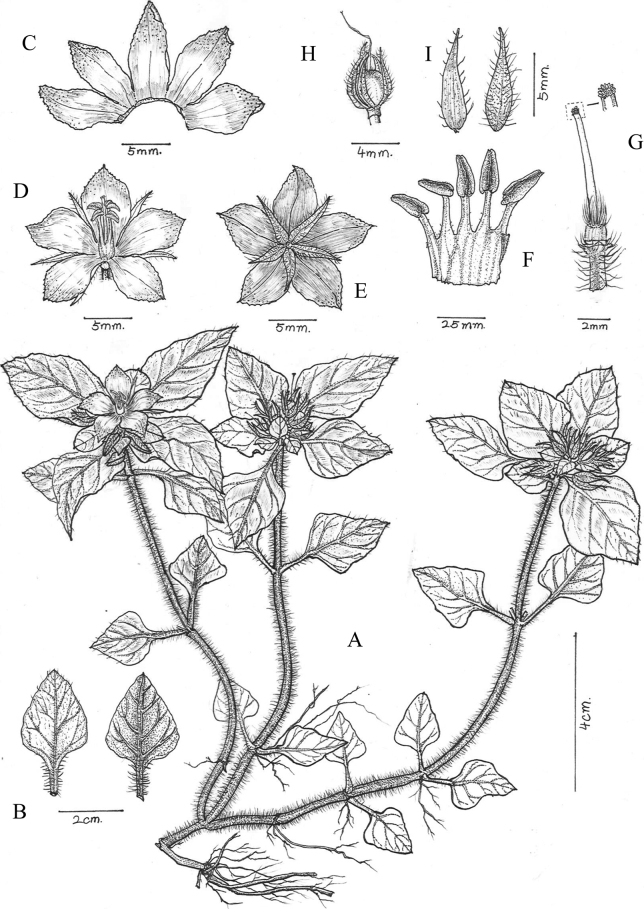
*Lysimachiadanxiashanensis* W.B.Liao, Q.Fan & G.D.Tang, sp. nov. **A** habit **B** abaxial (left) and adaxial (right) views of leaf **C** adaxial side of corolla lobes **D** lateral view of flower **E** dorsal view of flower **F** stamens **G** pistil and stigma **H** fruit **I** adaxial (left) and abaxial (right) views of calyx (Drawn by Rong-En Wu).

**Figure 4.**
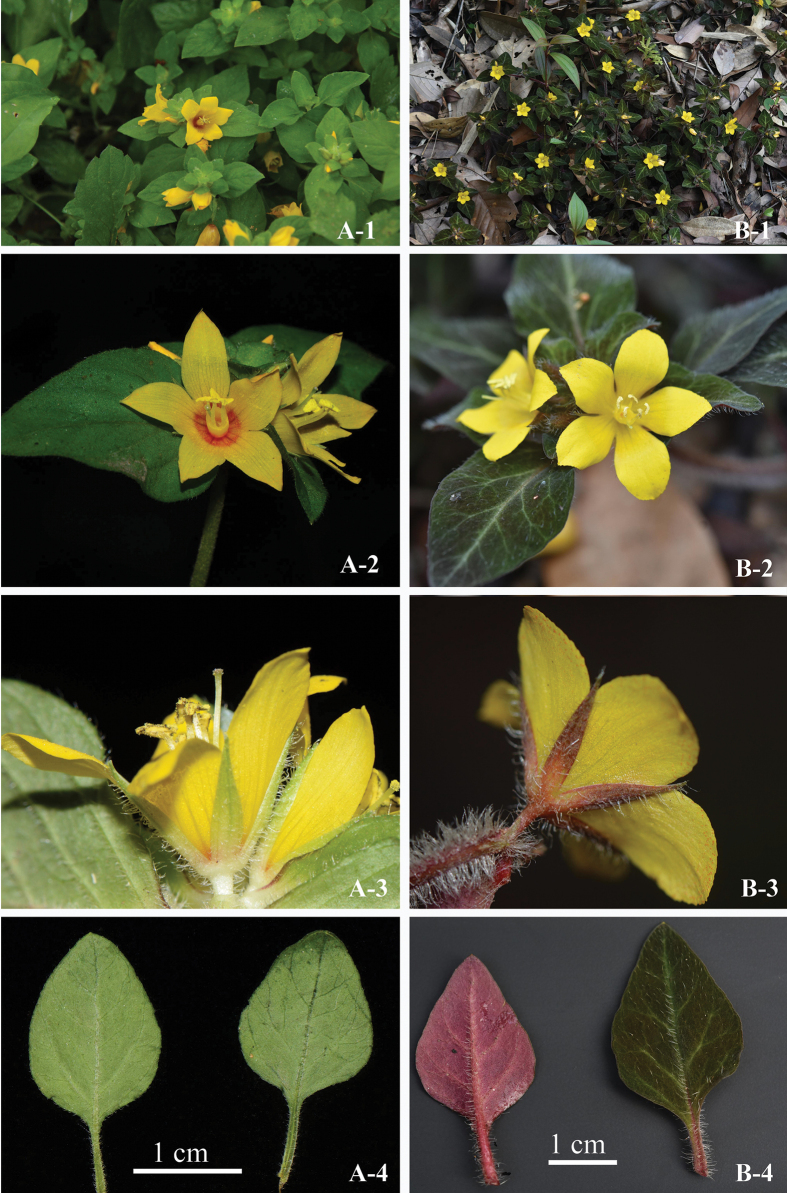
Morphological differences between *L.congestiflora* and *L.danxiashanensis***A***L.congestiflora***B***L.danxiashanensis***1** plants **2** corolla lobes **3** calyx lobes **4** petiole (Photographers: **A–1** by Wan-Yi Zhao **A2–4** by Xin-Xin Zhu **B1, 2, 4** by Xing-Yue Zhang **B–3** by Qiang Fan).

#### Type.

China. Guangdong Province, Danxiashan National Park, 25°0'N, 113°37'E, 311 m a.s.l., 12 May 2023 (fl.), *Xing-Yue Zhang, Zai-Xiong Chen* DNPC 3801 (holotype SYS!; isotypes CANT! SYS!).

#### Description.

Perennial herb, 10–28 cm tall. ***Stems*** prostrate, rooting at nodes, upper part ascending, purplish-red, terete, with dense white multicellular hairs. ***Leaves*** opposite, upper 2 or 3 pairs usually crowded, papery, ovate to broadly ovate or oval, wavy margin, 1.6–3.8 × 1.2–2.4 cm, apex acute, base broadly cuneate; adaxial surface dark green with antrorse strigose, abaxial surface purple-red, with strigose and densely pilose along the mid-rib vein; lateral veins 2–4-paired; petiole without wings, 0.5–2.7 cm, densely villous. ***Racemes*** terminal, abbreviated, capitate, 5–9 flowered; pedicel 3–5 mm long, densely pilose. ***Calyx*** 5, parted nearly to base; lobes lanceolate, 2–2.5 × 7–8.5 mm long, sparsely orange glandular on both surfaces, pilose outside. ***Corolla*** yellow; tube 1.5–2 mm; lobes 5, obovate-elliptical, 3–5 × 9–10 mm, serrate on upper half, apex acute to obtuse, abaxially glabrous, sparsely orange-red glandular, adaxially glabrous with orange-reddish glandular. ***Stamens*** 5, filaments glabrous, connate basally into a 2–3 mm high tube, free parts 2.8–4.5 mm; anthers ovate-lanceolate, dorsifixed, opening by lateral slits, ca. 2.8 mm long. ***Ovary*** white, terete, apex puberulous, glabrous at lower part; style glabrous, 6–8 mm long, stigma obtuse with papillae. ***Capsule*** subglobose, green, apex puberulous, 3–5 mm in diam.

#### Phenology.

The flowering of *Lysimachiadanxiashanensis* is from May to June; and the fruiting in June.

#### Distribution.

*Lysimachiadanxiashanensis* is currently known only from the type locality, Danxiashan National Nature Reserve, Guangdong, China.

#### Habit.

*Lysimachiadanxiashanensis* was observed to grow on wet rocks of Danxia landform at elevations 270 to 320 m.

#### Etymology.

The specific epithet refers to the type locality, Danxiashan National Nature Reserve in Guangdong Province, China.

#### Local name.

The Chinese name of the new species is here given as 丹霞山过路黄 (Dān xiá Shān Guò Lù Huáng).

#### Provisional conservation status.

Endangered (EN). In the past two years, we have conducted several field investigations on the Danxia landscapes in Guangdong Province, with only four populations of *Lysimachiadanxiashanensis* being found in Danxiashan National Nature Reserve and the number of mature individuals is stable (total < 200 individuals); However, its habitat is on the side of the road, which is vulnerable to human disturbance. *L.danxiashanensis* is regarded as Endangered (EN) according to D (the number of mature individuals in the population < 250) (IUCN Standards and Petitions Committee 2022).

#### Additional specimens examined

**(*paratypes*).** China, Guangdong: Danxiashan National Park, 25°0'N, 113°38'E, 298 m a.s.l., 12 June 2023 (fr.), *Jie-Hao Jin* DNPC 3803 (SYS!); Danxiashan National Park, 25°0'N, 113°38'E, 14 August 2023, *Qiang Fan, Jie-Hao Jin & Li-Juan Liao* DNPC 3845 (SYS!).

## Supplementary Material

XML Treatment for Lysimachia danxiashanensis

## ﻿Additional information

### Author ORCIDs

Xing-Yue Zhang https://orcid.org/0009-0007-5398-7100

Jing-Min Dai https://orcid.org/0009-0002-1090-4823

Qiang Fan https://orcid.org/0000-0003-4254-6936

Zai-Xiong Chen https://orcid.org/0009-0002-0475-6414

Guang-Da Tang https://orcid.org/0000-0001-5623-3928

Wen-Bo Liao https://orcid.org/0000-0001-6839-9511

### Data availability

The newly-obtained sequences of Lysimachiadanxiashanensis and L.kwangtungensis have been submitted to the NCBI website. The ITS and rbcL sequence data supporting the findings of this study are available within Appendix 1. The morphological data used in the study are included in this paper.

## ﻿Appendix 1

**Table A1.** List of the GenBank accession numbers of the *rbc*L and ITS sequences of sampled species in this study.

Taxon	Voucher	Locality	*rbc*L	ITS
*Lysimachiaalfredii* Hance	Hao394	Lianping, Guangdong, China	JF942344	JN638405
	Y2009279	Ruyuan, Guangdong, China	JF942343	JN638406
*Lysimachiacandida* Lindl.	Ge2010001	Yangchun, Guangdong, China	JF942346	JF976885
	Y2010016	Tongbai, Henan, China	JF942345	JF976884
*Lysimachiachapaensis* Merrill	GBOWS704	Maguan, Yunan, China	JF942350	JF976889
	GBOWS878	Hekou, Yunnan, China	JF942349	JF976888
	Hao209	Wuhan, Hubei, China	JF942392	AF547691
*Lysimachiachekiangensis* C.C.Wu	Y2009263-1	Longquan, Zhejiang, China	JF942352	JF976891
	Y2009263-2	Longquan, Zhejiang, China	JF942351	JF976890
*Lysimachiachristinae* Hance	Y2009209	Jiujiang, Jiangxi, China	JF942357	JF976896
	Y2009235	Shucheng, Anhui, China	JF942356	JF976895
	Y2009272	Jiangle, Fujian, China	JF942354	JF976893
*Lysimachiaclethroides* Duby	Y2009157	Tongbai, Henan, China	JF942362	JF976899
	Y2009248	Lin’an, Zhejiang, China	JF942360	JF976898
	Hao955	Wuxi, Chongqing, China	JF942359	JF976897
*Lysimachiacongestiflora* Hemsl.	Y2009196	Xinjian, Jiangxi, China	JF942367	JF976904
	Y2009266	Longquan, Zhejiang, China	JF942366	JF976903
	GBOWS262	Malipo, Yunnan, China	JF942365	JF976902
*Lysimachiacrispidens* Hemsl.	Hao212	Yichang, Hubei, China	JF942369	JF976906
	Y2010029	Xinhua, Hubei, China	JF942368	JF976905
*Lysimachiadecurrens* Forst.F.	GBOWS1234	Hekou, Yunnan, China	JF942371	JF976908
	Ye et al. 3980	Lianshan, Guangdong, China	JF942370	JF976907
Lysimachiadeltoideavar.cinerascens Franch.	Hao & Yan1033	Dali, Yunnan, China	JF942374	JF976911
	Hao731	Yongsheng, Yunnan, China	JF942373	JF976910
	GLM081121	Zhongdian, Yunnan, China	JF942372	JF976909
*Lysimachiadextrorsiflora* X.P.Zhang, X.H.Guo & J.W.Shao	Y2009265-1	Longquan, Zhejiang, China	JF942376	JF976913
	Y2009265-2	Longquan, Zhejiang, China	JF942375	JF976912
*Lysimachiaerosipetala* Chen et C.M.Hu	Y2010037-1	Emeishan, Sichuan, China	JF942378	JF976915
	Y2010037-2	Emeishan, Sichuan, China	JF942377	JF976914
Lysimachiafistulosavar.wulingensis Chen et C.M.Hu	Ye et al. 3561	Lianshan, Guangdong, China	JF942381	JF976917
	Ning20101	Jinggangshan, Jiangxi, China	JF942380	JF976916
*Lysimachiafordiana* Oliv.	Ye et al. 3940	Lianshan, Guangdong, China	JF942384	JF976920
*Lysimachiafortunei* Maxim	Y2009285	Ruyuan, Guangdong, China	JF942383	JF976919
*Lysimachiahemsleyana* Maxim	Guo20001	Ningguo, Anhui, China	JF942398	JF976932
	Y2009245	Lin’an, Zhejiang, China	JF942395	JF976929
	Y2010008	Tongbai, Henan, China	JF942394	JF976928
*Lysimachiahemsleyi* Franch.	Hao713	Huili, Sichuan, China	JF942402	JF976935
	Hao730	Yongsheng, Yunnan, China	JF942401	JF976934
*Lysimachiaheterogenea* Klatt	Y2009199	Jiujiang, Jiangxi, China	JF942407	JF976939
	Y2010009	Tongbai, Henan, China	JF942405	JF976938
*Lysimachiaklattiana* Hance	Y2010014-1	Tongbai, Henan, China	JF942415	JF976947
	Y2010014-2	Tongbai, Henan, China	JF942414	JF976946
*Lysimachialobelioides* Wall.	Hao303	Menglian, Yunnan, China	JF942419	JF976951
	Y2010001	Jingping, Yunan, China	JF942418	JF976950
*Lysimachialongipes* Hemsl.	Y2009255-1	Kaihua, Zhejiang, China	JF942422	JF976954
	Y2009255-2	Kaihua, Zhejiang, China	JF942421	JF976953
	Guo xinhu200012	Shitai, Anhui, China	JF942420	JF976952
*Lysimachiamelampyroides* R.Knuth	Lichanghan8174	Shangzhi, Hunan, China	JF942424	JF976956
	Dengyunfei15945	Xinning, Hunan, China	JF942423	JF976955
*Lysimachiaomeiensis* Hemsl.	Y2010033	Emeishan, Sichuan, China	JF942426	JF976958
	Hao224	Emeishan, Sichuan, China	JF942425	JF976957
Lysimachiaparidiformisvar.paridiformis Franch.	Chen s.n.	Enshi, Hubei, China	JF942429	JF976961
Lysimachiaparidiformisvar.stenophylla Franch.	Deng15921	Xinning, Hunan, China	JF942431	JF976963
	Y2010044	Emeishan, Sichuan, China	JF942430	JF976962
	GLM07658	Zhenxiong, Yunnan, China	JF942428	JF976960
*Lysimachiapatungensis* Hand.-Mazz.	Y2009187	Jinggangshan, Jiangxi, China	JF942435	JF976967
	Y2009258	Kaihua, Zhejiang, China	JF942434	JF976966
	Y2009280	Ruyuan, Guangdong, China	JF942433	JF976965
	Ye et al. 3851	Lianshan, Guangdong, China	JF942432	JF976964
*Lysimachiapentapetala* Bunge	Y2010013-1	Tongbai, Henan, China	JF942437	JN638407
*Lysimachiaphyllocephala* Hand.-Mazz.	Y2010030	Emeishan, Sichuan, China	JF942439	JF976969
	Y2010048	Nanchuan, Chongqing, China	JF942438	JF976968
	GLM07662	Yanjin, Yunnan, China	JF942399	JF976933
*Lysimachiapseudohenryi* Pamp.	Guo XH 20007	East Asia	MG950600	MG877828
*Lysimachiarubiginosa* Hemsl.	Hao704	Hongya, Sichuan, China	JF942444	JF976974
	Y2010036	Emeishan, Sichuan, China	JF942443	JF976973
	Hao419	Dujiangyan, Sichuan, China	JF942442	JF976972
*Lysimachiadanxiashanensis*	DNPC-3711	Danxiashan, Guangdong, China	PP025352	OR665389
	DNPC-3711	Danxiashan, Guangdong, China	PP025354	OR665390
*Lysimachiakwangtungensis* (Handel-Mazzetti) C.M.Hu	DNPC-3743	Danxiashan, Guangdong, China	PP025355	OR941025
*Ardisiaverbascifolia* Mez.	GBOWS1216	Hekou, Yunnan, China	JN638410	JN638408
